# Editorial: Combination Strategies in the Treatment of Melanoma

**DOI:** 10.3389/fonc.2016.00067

**Published:** 2016-03-22

**Authors:** Paolo Antonio Ascierto

**Affiliations:** ^1^Istituto Nazionale Tumori Fondazione “G. Pascale”, Naples, Italy

**Keywords:** melanoma, immunotherapy, vasculopathy, inhibitors, angiogenesis inhibitors

**The Editorial on the Research Topic**

**Combination Strategies in the Treatment of Melanoma**

The successful treatment of melanoma was considered a significant challenge by oncologists for many years. However, this situation changed in 2011 with the approval of two novel agents, the anti-CTLA4 monoclonal antibody, ipilimumab, and the BRAF inhibitor, vemurafenib. These two drugs completely changed the management of metastatic melanoma and, for the first time, it became possible to significantly improve overall survival. More new therapies have followed, including other targeted agents (e.g., MEK inhibitors) and immunotherapies (e.g., anti-PD-1 agents). The availability of these new agents has resulted in a focus on combination strategies aimed at improving outcomes still further. Novel combinations of targeted drugs and/or immunotherapies represent the future treatment not only for melanoma but also for all solid tumors. The articles collected together in this Research Topic discuss various aspects of the use combination strategies in melanoma management.

Inhibition of angiogenesis through the targeting of vascular endothelial growth factor (VEGF) has been used in the treatment of cancer for many years, although the mechanisms of its antitumor activity remain poorly understood. More recently, the advent of immune checkpoint inhibition (e.g., ipilimumab and nivolumab) has led to a better understanding of the possible effects of immunotherapy on immune-mediated vasculopathy in the tumor, with the suggestion that the tumor vasculature may be an important interface between the tumor-directed immune response and the cancer itself. Initial studies of the relationship between angiogenesis, VEGF signaling and the immune system suggest that the combination of immune checkpoint blockade with VEGF inhibition has potential. The rationale for combined angiogenesis inhibition and immune checkpoint inhibition is reviewed by Ott et al. Clinical studies of combined immunotherapy and VEGF inhibition, e.g., ipilimumab plus bevacizumab, are ongoing and represent a potentially promising approach for cancer treatment.

The use of combined BRAF and MEK inhibition in advanced melanoma in order to overcome resistance and reduce toxicity is reviewed by McArthur, who notes that the impact of pathway inhibition in the adjuvant setting, where there can be differences in the extent of response of micro-metastases or in the microenvironment that emerges following treatment, necessitates ongoing preclinical and clinical research into therapeutic targeting of the pathway. Combination approaches that look at simultaneous or sequential use of immunotherapeutic approaches with agents that target the RAF/MEK/ERK pathway are also a priority, with evidence suggesting that targeted therapies can sensitize tumor cells to immune attacks and improve the effector function of immune cells, but it should not divert attention away from the pathway that induces such profound oncogene addiction in melanoma patients whose tumors contain activating mutations in BRAF.

New treatment strategies that target key effectors of the RAF/MEK/ERK pathway may display intrinsic or acquired resistance. Evidence suggests that inhibition of a single effector involved in melanoma pathogenesis may be ineffective in blocking tumor growth. As such, a better understanding of the multiple molecular alterations accounting for either response or resistance to treatments with targeted inhibitors may be useful in identifying which combination strategies are likely to be the most effective. In their paper, Palmieri et al. summarize the known molecular mechanisms underlying intrinsic and acquired drug resistance and highlight the need for strategies that combine the most effective targeted treatments with cancer immunotherapies.

Resistance to BRAF and MEK inhibitors can be mediated by activation of the PI3K pathway, providing a strong rationale for combination therapy in melanoma. Original research by Sweetlove et al. investigated the effects of a MEK inhibitor (selumetinib)and a BRAF inhibitor (vemurafenib) alone and in combination with inhibitors of pan-PI3K (ZSTK474), pan-PI3K/mTOR (BEZ235), individual PI3K isoforms (p110α, A66; p110β, TGX-221; p110γ, AS-252424; p110δ, idelalisib), or mTORC1/2 (KU-0063794) on nine metastatic BRAF-mutated melanoma cell lines. Selumetinib and vemurafenib potently inhibited cell proliferation in all cell lines, especially in those that expressed low levels of phosphorylated AKT (pAKT). Pan-PI3K and pan-PI3K/mTOR inhibitors also inhibited cell proliferation in all cell lines and enhanced the antitumor activity of selumetinib and vemurafenib in the majority of lines. These results suggest that the sensitivity of BRAF-mutant melanoma cells to BRAF or MEK inhibitors is at least partly mediated by activation of the PI3K pathway and can be enhanced by combined inhibition of the BRAF/MEK and PI3K/mTOR signaling pathways.

Neuropilin-1 (NRP-1) is a transmembrane glycoprotein that acts as a coreceptor for various members of the VEGF family. Its ability to bind or modulate the activity of a number of other extracellular ligands has suggested involvement in a variety of physiological and pathological processes. NRP-1 is also expressed in a variety of cancers, suggesting a critical role in tumor progression. Increased levels of NRP-1 are correlated to tumor aggressiveness and are associated with poor prognosis. Graziani and Lacal review the role of NRP-1 in melanoma aggressiveness and the evidence supporting its use as target of therapies for metastatic melanoma.

An overview of the main immuno-oncology treatment strategies that, either alone or in combination, are undergoing clinical development is provided by Ascierto et al. These include immunotherapeutic strategies that include adoptive transfer of *ex vivo* activated T cells, immunomodulatory monoclonal antibodies, and cancer vaccines. These focus on approaches being investigated in the treatment of melanoma, which has been transformed by immunotherapy into a potentially curable disease.

## Author Contributions

The author confirms being the sole contributor of this work and approved it for publication.

## Conflict of Interest Statement

The author declares that the research was conducted in the absence of any commercial or financial relationships that could be construed as a potential conflict of interest.

